# Potato ingestion is as effective as carbohydrate gels to support prolonged cycling performance

**DOI:** 10.1152/japplphysiol.00567.2019

**Published:** 2019-10-17

**Authors:** Amadeo F. Salvador, Colleen F. McKenna, Rafael A. Alamilla, Ryan M. T. Cloud, Alexander R. Keeble, Adriana Miltko, Susannah E. Scaroni, Joseph W. Beals, Alexander V. Ulanov, Ryan N. Dilger, Laura L. Bauer, Elizabeth M. Broad, Nicholas A. Burd

**Affiliations:** ^1^Department of Kinesiology and Community Health, University of Illinois, Urbana, Illinois; ^2^Division of Nutritional Sciences, University of Illinois, Urbana, Illinois; ^3^Roy J. Carver Biotechnology Center, Urbana, Illinois; ^4^Department of Animal Sciences, University of Illinois, Urbana, Illinois; ^5^Beckman Institute for Advanced Science and Technology, University of Illinois, Urbana, Illinois; ^6^US Olympic and Paralympic Committee, Chula Vista, California

**Keywords:** carbohydrate, endurance, exercise, sports nutrition

## Abstract

Carbohydrate (CHO) ingestion is an established strategy to improve endurance performance. Race fuels should not only sustain performance but also be readily digested and absorbed. Potatoes are a whole-food-based option that fulfills these criteria, yet their impact on performance remains unexamined. We investigated the effects of potato purée ingestion during prolonged cycling on subsequent performance vs. commercial CHO gel or a water-only condition. Twelve cyclists (70.7 ± 7.7 kg, 173 ± 8 cm, 31 ± 9 yr, 22 ± 5.1% body fat; means ± SD) with average peak oxygen consumption (V̇o_2peak_) of 60.7 ± 9.0 mL·kg^−1^·min^−1^ performed a 2-h cycling challenge (60–85% V̇o_2peak_) followed by a time trial (TT; 6 kJ/kg body mass) while consuming potato, gel, or water in a randomized-crossover design. The race fuels were administered with [U-^13^C_6_]glucose for an indirect estimate of gastric emptying rate. Blood samples were collected throughout the trials. Blood glucose concentrations were higher (*P* < 0.001) in potato and gel conditions compared with water condition. Blood lactate concentrations were higher (*P* = 0.001) after the TT completion in both CHO conditions compared with water condition. TT performance was improved (*P* = 0.032) in both potato (33.0 ± 4.5 min) and gel (33.0 ± 4.2 min) conditions compared with water condition (39.5 ± 7.9 min). Moreover, no difference was observed in TT performance between CHO conditions (*P* = 1.00). In conclusion, potato and gel ingestion equally sustained blood glucose concentrations and TT performance. Our results support the effective use of potatoes to support race performance for trained cyclists.

**NEW & NOTEWORTHY** The ingestion of concentrated carbohydrate gels during prolonged exercise has been shown to promote carbohydrate availability and improve exercise performance. Our study aim was to expand and diversify race fueling menus for athletes by providing an evidence-based whole-food alternative to the routine ingestion of gels during training and competition. Our work shows that russet potato ingestion during prolonged cycling is as effective as carbohydrate gels to support exercise performance in trained athletes.

## INTRODUCTION

Carbohydrate (CHO) ingestion during prolonged endurance exercise (>2 h) is a proven dietary strategy to sustain exercise performance (31). The factors that contribute to the increased exercise performance with CHO ingestion include maintenance of blood glucose concentrations, high exogenous CHO oxidation rates during the late stages of a race, and attenuation in the decline of liver glycogen during prolonged exercise (18). Indeed, the amount of ingested CHO required to support exercise performance is closely connected to the intensity and duration of the exercise bout, but recommendations generally range from 30 to 60 g/h, with some recommendations as high as 90 g/h depending on the type of CHO consumed and duration of exercise (24).

Specifically formulated sports foods, such as concentrated CHO gels, are commonly used by endurance athletes to enhance CHO availability during training and competition (19). The form (e.g., liquid vs. solid) in which the CHO is ingested does not appear to modulate its delivery and oxidation during exercise (32, 35). Hence, optimal race feeding is somewhat personalized, and race fuel selection will depend on a variety of factors, including taste, cost, and the risk of gastrointestinal (GI) distress. The latter is pertinent, since the prevalence of exercise-induced GI distress has been reported by 30–70% of endurance athletes (6, 12), and this GI distress may negatively impact their performance (12). As such, the gut has been increasingly recognized as an athletic organ (25); therefore, the most appropriate race fuel should facilitate gastric emptying and intestinal absorption and deliver targeted amounts of exogenous CHO without exacerbating GI symptoms (e.g., cramping, bloating, vomiting, etc.) during competition (23).

While commercially available sports foods have been shown to effectively increase exercise performance (7), it is relevant to identify other high-performance foods to provide diet (CHO) diversity for an athlete. Therefore, the purpose of the present study was to assess the effectiveness of potato ingestion as a fueling strategy to support cycling time trial (TT) performance compared with CHO gel or water in trained cyclists. Potatoes are a promising alternative for athletes because they represent a cost-effective, nutrient-dense, whole-food source of CHO; furthermore, they serve as a savory race fuel option compared with the high sweetness of CHO gels. We examined other relevant variables that might be related to exercise performance and nutrient bioavailability, such as symptoms of GI discomfort, plasma intestinal fatty acid-binding protein concentrations (I-FABP; a marker of small intestine injury), and core temperature (i.e., impact of exogenous CHO source on thermoregulatory capacity). Finally, [U-^13^C_6_]glucose was orally administered to provide (indirect) insight into the appearance rate of ingested glucose into the circulation. We hypothesized that potato and gels ingested at 60 g CHO/h during a 2-h cycling challenge would be more effective on subsequent cycling TT performance than consuming only water in trained cyclists.

## METHODS

### 

#### Participants and ethical approval.

Twelve cyclists (*n* = 9 male, *n* = 3 female; 70.7 ± 7.7 kg, 173 ± 8 cm, 30.6 ± 8.7 yr, 21.6 ± 5.1% body fat) volunteered to participate in this study. Participants cycled on average 267 km/wk (range 120–480 km/wk) and had been training an average of 7 yr (range 3–20 yr). Based on peak oxygen consumption (V̇o_2peak_, 60.7 ± 9.0 mL·kg^−1^·min^−1^), peak workload (W_peak_, 350 ± 63 W), and W_peak_/kg (4.9 ± 0.7 W/kg), the participants were classified as endurance trained and competitive (13). Experimental trials were completed during the midfollicular phase of the menstrual cycle for the female participants. All participants were considered healthy on the basis of a self-reported medical screening questionnaire. Each participant was informed of the purpose of the study, the experimental procedures, and all of the potential risks before providing their written consent to participate. The study was approved by the University of Illinois Institutional Review Board and conformed to standards for the use of human participants in research as outlined in the *Declaration of Helsinki*. This trial is registered at https://www.clinicaltrials.gov as NCT03294642.

#### Pretesting.

All participants underwent pretesting procedures on two separate occasions. On the first visit, body weight, height, and body composition by dual-energy X-ray absorptiometry (QDR 4500A; Hologic, Marlborough, MA) were measured. Subsequently, participants performed an incremental cycling test on an electronically braked cycle ergometer (Lode Excalibur Sport, Groningen, The Netherlands) with the initial power set at 2 W/kg body wt and increased by 30 W for males and 20 W for females every 1 min until exhaustion. V̇o_2peak_ was determined as the highest recorded 20-s V̇o_2_ value when at least three criteria were satisfied: *1*) a plateau in V̇o_2_ despite an increase in work rate, *2*) respiratory exchange ratio ≥1.10, *3*) heart rate peak within 10 beats/min of age-predicted maximum (i.e., 220-age), or *4*) ratings of perceived exertion (RPE; Borg scale 6–20) ≥ 17. The V̇o_2peak_ workload (V̇o_2ppeak_) and peak workload (W_peak_) were defined as the intensity related to the V̇o_2peak_ and the final intensity achieved at the end of the test, respectively. Inclusion criteria were set at the minimum V̇o_2peak_ of 50 mL·kg^−1^·min^−1^ for males and 45 mL·kg^−1^·min^−1^ for females. During the screening phase, four participants were excluded for not meeting this threshold; however, the 12 participants enrolled achieved a V̇o_2peak_ above the minimum threshold. The participant’s preferred cadence was also determined during incremental test, with first and last stages excluded from the calculation to avoid ergometer adaptation and fatigue effect, respectively. The seat position was recorded and replicated for all the subsequent tests.

On the second visit to the laboratory, participants performed a familiarization ride consisting of a 120-min cycling challenge followed by a TT. The prescribed cycling challenge intensities were predicted based on the incremental test and confirmed based on respiratory gases collected during the first hour of the familiarization ride. During this trial, the participants used their own preferred fueling strategy. Participants were excluded if they were not able to complete the cycling challenge or the TT. During the screening phase, two participants were excluded because they could not complete the familiarization trial. The 12 participants studied successfully completed the familiarization trial. Afterward, participants were randomized with the trial order counterbalanced to consume either *1*) baked white potato flesh purée (60 g CHO/h), *2*) commercially-available energy gel (60 g CHO/h), or *3*) water.

#### Dietary and activity control.

Exercise and nutritional statuses were controlled before each experimental trial. Specifically, participants consumed standardized meals provided by the research team for 24 h before each experimental trial. The meal plans were designed by a registered dietitian to mimic recommended nutritional practices for endurance sport. Specifically, each meal had an energy content of 9 kcal/kg body mass and was composed of 60% CHO [1.4 g·kg^−1^·meal^−1^ (7 g·kg^−1^·day^−1^)], 20% protein (0.4 g·kg^−1^·meal^−1^), and 20% fat (0.2 g·kg^−1^·meal^−1^), with breakfast, lunch, dinner, and two snacks being the mealtimes emphasized. Consumed meals were recorded and replicated for the next trials. In addition, the participants were requested to abstain from drinking alcohol for 48 h and from ingesting caffeine and/or NSAIDs (nonsteroidal anti-inflammatory drugs) the morning of their experimental trials. Participants were also provided with an ingestible thermistor capsule (HQ, Inc., Palmetto, FL) to be consumed 8–12 h before the experimental trials. Diet and training diaries were used to assess compliance and were returned to allow the participant to repeat identical habits before each trial. In addition, the participants were requested to avoid any type of exercise 48 h before the trials.

#### Experimental protocol.

Each participant arrived at the laboratory at the same time in the morning after an overnight fast. On arrival, an intravenous catheter was inserted into an antecubital vein and kept patent with 0.9% saline drip for repeated blood sampling. After baseline blood sampling, participants were provided with a standardized breakfast (1 g/kg CHO, 0.4 g/kg protein) with water provided ad libitum. Participants rested in the laboratory for 2 h before the commencement of the cycling challenge. Before the cycling challenge, participants provided a urine sample to determine baseline urine osmolality and urine specific gravity (USG; osmometer model 3320, Advanced Instruments, Norwood, MA) and were towel dried before preexercise weight measurements.

**Fig. 1.**
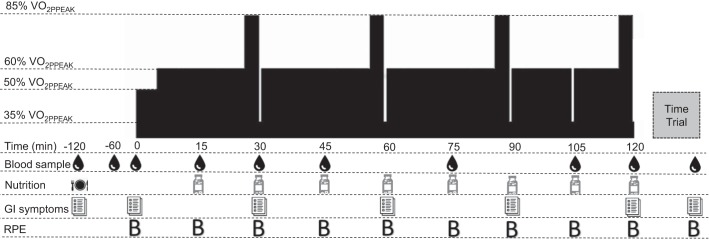
Overview of experimental design. A postabsorptive blood sample was obtained before ingestion of a standardized breakfast (−120 min). The cycling challenge (0–120 min, 60% V̇o_2ppeak_) was initiated with a 5-min warm-up (50% V̇o_2peak_), with hills (85% V̇o_2peak_/3 min) followed by downhills (35% V̇o_2ppeak_/1 min) every 30 min. A downhill at 105 min allowed for mask placement to collect gas exchange. A 6 kJ/kg time trial was initiated after cycling challenge completion. V̇o_2ppeak_, peak O_2_ consumption workload; GI, gastrointestinal; RPE, rate of perceived exertion (Borg scale, 6–20).

The exercise protocol consisted of a 120-min cycling challenge immediately followed by a TT (6 kJ/kg body mass) completed as fast as possible. As shown in Fig. 1, the cycling challenge started with a 5-min warm-up at 50% V̇o_2peak_ followed by steady-state exercise at 60% V̇o_2peak_, with four intermittent, high-intensity bursts (each 3 min at 85% V̇o_2peak_) to simulate hill climbs. Each burst was immediately followed by a low-intensity period (1 min at 35% V̇o_2peak_) to simulate descents. “Hills” and “descents” were performed once every 30 min. On two of the trials, participants were administered supplemental CHO (15 g of CHO administered every 15 min) in the form of baked russet potato flesh purée (128.5 g per bolus) or CHO gels (PowerBar; 23 g per bolus). All treatments were supplemented with 2% enriched (0.3 g) [U-^13^C_6_]glucose to provide a proxy for gastric emptying rates and the subsequent appearance of exogenous glucose into circulation (3). Blood sampling, heart rate, core temperature, RPE (Borg scale 6–20), and GI symptoms were assessed throughout the cycling challenge as indicated in Fig. 1. For the TT, the ergometer was set in linear mode with the linear factor based on their personal 70% P_peak_ and preferred cycling cadence determined during the incremental test. In this ergometer mode, an increase in cadence resulted in an equivalent increase in the required workload. During the TT, encouragement was withheld until the last 10% of the TT and no information about performance was provided. After completion of the TT, participants were towel dried and weighed, and subsequently provided a urine sample. For the RPE analysis of the TT, we adopted a ratio of RPE by workload, as previously described (16). This calculation accounts for the Borg scale’s ceiling effect (4). The GI symptoms (i.e., overall symptoms, abdominal pain, abdominal bloating, gut rumbling, flatulence, abdominal discomfort) were rated against a standardized 0- to 100-mm visual analog scale (VAS) questionnaire. Blood samples were collected in EDTA-containing tubes and centrifuged at 3,000  *g* at 4°C for 10 min. Aliquots of plasma were frozen and stored at −80°C until subsequent analysis.

#### Race fuel preparation and analysis.

Russet potatoes were purchased fresh before each trial. Potatoes were microwaved, peeled, and blended in a food processor, and then both processed potatoes and gel were analyzed for total nonstructural carbohydrate content (gross measure of the proportion of CHO that could be digested by mammalian enzymes) (38) to determine appropriate serving size for goal CHO dose. 548 g of potato flesh (~1.1 kg raw potatoes), baked in skin, yielded 120 g of CHO for the 2-h cycling challenge. The baked-in-skin potato flesh was blended with 478.5 mL of H_2_O and 2.4 g of table salt (NaCl) to achieve a consistency and salinity similar to those of the CHO gels. Sport gels of 184 g, 120 g CHO; PowerBar, Power Gel, vanilla; Premier Nutrition, Emeryville, CA) were consumed during the respective cycling challenge. Both the potato purée and the gels were aliquoted into eight servings (15 g CHO), and refrigerated (4°C) until trial day time of ingestion. CHO conditions were administered in 30-mL disposable syringes to standardize method of delivery. The gels did not contain caffeine or any stimulants.

An additional aliquot of CHO conditions (potato and gel) from each trial was frozen at −20°C for future CHO compositional analysis. Samples were freeze-dried and ground, and a subsample was heated to 105°C to determine dry matter content; all subsequent extractions were calculated on a dry matter basis. Crude protein, ash, and total dietary fiber (along with an insoluble/soluble split) were extracted by α-amylase and amyloglucosidase as previously described (36). Free monosaccharides, oligosaccharides, and fructooligosaccharides were isolated by high-performance liquid chromatography (HPLC) analysis (8, 37). No quantifiable amounts of fructo- and galactooligosaccharides were detected in either potato or gel samples. Monosaccharides and sugar alcohols were extracted by sulfuric acid hydrolysis with 2-deoxyglucose added as an internal standard. Composition was determined by HPLC analysis and quantified against known standards of various monosaccharides and sugar alcohols (21). The nutrient composition and estimated energy yields (44) of the treatments are shown in Table 1.

**Table 1.** Nutrient composition of treatment conditions

Nutrient	Potato	Gel
Carbohydrate dose, g	15.2	15.5
Total serving size, g	1,028	184
Moisture content, %	86	32
Energy, kcal	548	494
Crude protein, g	13.9	0.1
Total carbohydrate, g	121.3	123.7
Total dietary fiber	11.2	2.3
Soluble fiber	6.6	2.3
Insoluble fiber	4.6	0.0
Hydrolyzed monosaccharides	129.8	129.4
Total glucose	120.5	90.4
Total galactose	3.9	0.0
Total fructose	4.3	39.0

Carbohydrate dose administered every 15 min for 2 h. Total serving size expressed on an as-is basis. Sample aliquots were dried to completion at 105°C to determine dry-matter content (i.e., non-water portion of the original sample). All nutrients were analyzed and their composition values calculated and expressed on a dry-matter basis (DMB) to ensure an equal comparison between potato and gel samples. Energy estimated from USDA Database, based on the Atwater system (44). Total carbohydrate calculated by difference (organic matter − crude protein) in DMB.

Fluid intake was also controlled during all three trials. *Experimental trial 1*, irrespective of condition, served to identify each participant’s “usual” water intake by allowing water ad libitum. This amount was recorded and replicated throughout all subsequent trials. Water used for potato purée preparation was accounted for in the total water allowance. We used this approach since fluid intake guidelines are varied and highly individualized in trained athletes due to differential sweat rates. Hydration status was assessed from pre- to postexercise changes in body mass, urine osmolality, and USG.

#### Blood analysis.

Glucose and lactate were analyzed in whole blood by use of an automated biochemical analyzer (YSI 2300 Stat Plus; YSI, Yellow Springs, OH). Plasma insulin concentrations were determined with a commercially available enzyme-linked immunosorbent assay (ELISA) (ALPCO Diagnostics, Salem, NH) and expressed as area under the curve (AUC) during the cycling challenge. Plasma I-FABP concentrations were assessed with an ELISA according to the manufacturer’s instructions (Hycult Biotechnology, Uden, The Netherlands) and was expressed as fold change from baseline. Plasma [U-^13^C_6_]glucose enrichments were determined by gas chromatography-mass spectrometry (GC-MS) analysis (7890A GC/5975C MSD, Agilent). Briefly, plasma samples were deproteinized and converted into their *tert*-butyldimethylsilyl derivatives, and enrichments were determined using electron ionization by ion monitoring at *m/z* of 319 (*m*+0), 321 (*m*+2), and 323 (*m*+4). Plasma glucose enrichments for each labeled ion were expressed relative to 319 (*m*+0, tracee), and enrichment was expressed as tracer-to-tracee-ratio (TTR). All blood metabolites were analyzed blindly.

#### Expired gas analysis.

Oxygen consumption (V̇o_2_), carbon dioxide production (V̇co_2_), and ventilation per minute (VE) were measured breath by breath using an automated open-circuit gas analysis system (TrueOne 2400 Parvo Medics, Inc., Salt Lake City, UT) throughout each test. During the cycling challenge, the last 15 min of expired gas was collected, and 30-s averages between 111.5 and 116 min of the cycling challenge were used to calculate fat and CHO oxidation rates during exercise in a blinded-fashion according to the equations below (17):

$$\text{Fat oxidation}=\left(1.695\cdot \dot{\text{V}}{\text{O}}_{2}\right)–\left(1.701\cdot \dot{\text{V}}{\text{CO}}_{2}\right)$$

$$\text{Carbohydrate oxidation}=\left(4.21\cdot \dot{\text{V}}{\text{CO}}_{2}\right)–\left(2.962\cdot \dot{\text{V}}{\text{O}}_{2}\right)$$

with V̇o_2_ and V̇co_2_ in liters per min (L/min) and oxidation rates in grams per minute (g/min).

#### Statistics.

Based on a priori power analysis, twelve participants exceeded the minimum sample size required to detect a difference in TT performance with a power of 0.80. This power calculation was based on a two-tailed α-level of 0.05 and past efforts that had used a similar TT approach (42). The effect of nutritional strategy on outcomes was estimated via a linear mixed-model analyses of variance using the software SPSS v. 20. For analysis of plasma [U-^13^C_6_]glucose enrichments, glucose, lactate, insulin, I-FABP, CHO and fat oxidation, RPE, GI symptoms, workload, total work, and heart rate, the fixed factors were time and condition (water, potatoes, or gel), and the random factor was the subject. For analysis of TT performance, weight loss, USG, and urine osmolality condition was the only fixed factor. The TT was divided into four quartiles for performance, RPE, and heat rate analyses. Bonferroni’s post hoc tests were performed to determine differences between means for all significant main effects and interactions. To evaluate the relationship between TT performance and I-FABP or glucose concentrations at 120 min (onset of TT), the repeated-measures correlation analysis was performed using the rmcorr R package developed by Bakdash and Marusich (https://cran.r-project.org/web/packages/rmcorr/) (2). The level of statistical significance was set at *P* < 0.05 for all analysis. The data are expressed as means ± SD.

## RESULTS

### 

#### Challenge and time trial.

The average differences between experimental trial start time for cycling challenge and TT were 11 ± 10 and 9 ± 9 min, respectively. Total weight loss did not differ (*P* = 0.824) between water (−2.04 ± 0.89 kg), potato (−1.84 ± 0.74 kg), or gel conditions (−1.87 ± 0.59 kg). Similarly, USG was not different (*P* = 0.605) between the water (PRE, 1.008 ± 0.005; POST, 1.010 ± 0.004), potato (PRE, 1.011 ± 0.009; POST: 1.009 ± 0.004), and gel conditions (PRE, 1.011 ± 0.007; POST: 1.010 ± 0.004). Before the start of the cycling challenge, urine osmolalities were 323 ± 189, 380 ± 275, and 374 ± 260 mosmol/kg for water, potato, and gel conditions, respectively. After completion of the TT, urine osmolalities were 351 ± 158, 316 ± 150, and 341 ± 150 mosmol/kg for water, potato, and gel conditions, respectively. No time (*P* = 0.875) or condition (*P* = 0.740) effects were observed in urine osmolality.

The average absolute challenge intensities were 150 ± 32, 180 ± 30, 278 ± 50, and 93 ± 21 W for 50, 60, 85, and 35% V̇o_2ppeak_, respectively. These intensities represent 43 ± 4, 52 ± 2, 80 ± 5, and 27 ± 3% W_peak_, respectively. Average total work performed during the entire challenge was 1332 ± 232 kJ, and specifically 45 ± 9, 778 ± 133, 200 ± 36, and 28 ± 6 kJ at the intensities 50, 60, 85, and 35% V̇o_2ppeak_, respectively. Heart rate responses during the cycling challenge were not different between conditions (*P* = 0.962). The heart rate average values at the first, second, third, and fourth hills were 167 ± 8, 166 ± 8, 167 ± 9, and 169 ± 8 beats/min for the water condition, 167 ± 7, 168 ± 8, 167 ± 8, and 168 ± 8 beats/min for the potato condition, and 167 ± 8, 168 ± 8, 168 ± 7, and 169 ± 8 beats/min for the gel condition, respectively. During the TT, CHO ingestion, irrespective of condition, resulted in a higher percentages of peak heart rate obtained compared with water condition (*P* < 0.01). Peak heart rate was obtained during the incremental test. The percentage values for each condition were 86 ± 11, 86 ± 11, and 85 ± 10% (water); 91 ± 8, 90 ± 8, and 92 ± 8% (potato); and 91 ± 9, 91 ± 9, and 93 ± 7% (gel) during the second, third, and fourth quartiles of the TT, respectively.

#### Whole body substrate oxidation.

A main effect of condition was observed in CHO and fat oxidation (*P* < 0.001), with no effect of time (*P* = 1.00). Gel (1.79 ± 0.59 g/min, *P* < 0.001) and potato (1.69 ± 0.40 g/min, *P* < 0.001) conditions showed higher CHO oxidation than the water condition (1.42 ± 0.54 g/min). Similarly, fat oxidation was higher in water (0.75 ± 0.28 g/min) than in potato (0.65 ± 0.25 g/min, *P* = 0.017) and gel conditions (0.59 ± 0.26 g/min, *P* < 0.001). There was no difference between gel and potato conditions in CHO (*P* = 0.556) and fat oxidation (*P* = 0.437).

#### Rating of perceived exertion.

**Fig. 2.**
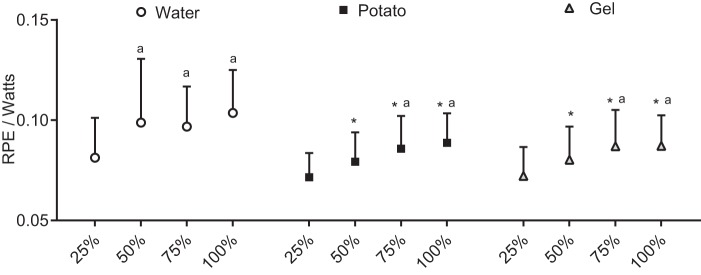
Ratings of perceived exertion (RPE) during the time trial relative to load. All values are presented as means ± SD (*n* = 12). *Significant difference from water condition (*P* < 0.01); ^a^significant difference from 25% within condition (*P* < 0.01).

RPE values at 60 min (water, 14.9 ± 2.1; potato, 14.6 ± 1.9; gel, 14.4 ± 2.5) and at the cessation of the cycling challenge (water, 17.5 ± 2.3; potato, 16.5 ± 2.4; gel, 17 ± 2) were different (*P* < 0.001) from baseline (water, 7.5 ± 1.7; potato, 7.0 ± 1.4; gel, 7.7 ± 1.9). No differences (*P* = 0.106) between conditions were observed in raw and fold change controlled by the baseline value. However, significant differences were observed in RPE relative to load performed between potato (*P* = 0.005) and gel (*P* = 0.008) conditions vs. water condition during the TT (Fig. 2).

#### Core temperature.

There was no difference in core temperature (*P* = 0.779) among conditions during the cycling challenge. The core temperature increased significantly from the beginning of the exercise in water (*P* = 0.003), potato (*P* = 0.037), and gel (*P* = 0.015) conditions at 24, 17, and 19 min, respectively. In addition, even with no differences (*P* = 0.685) in the baseline value among water (36.9 ± 0.3°C), potato (36.8 ± 0.3°C), and gel conditions (37.0 ± 0.4°C), core temperature value at the onset of the TT was lower (*P* = 0.045) in potato (37.8 ± 0.5°C) than in gel (38.3 ± 0.5°C) condition, with no differences compared with the water condition (37.9 ± 0.5°C).

#### Blood analysis.

**Fig. 3.**
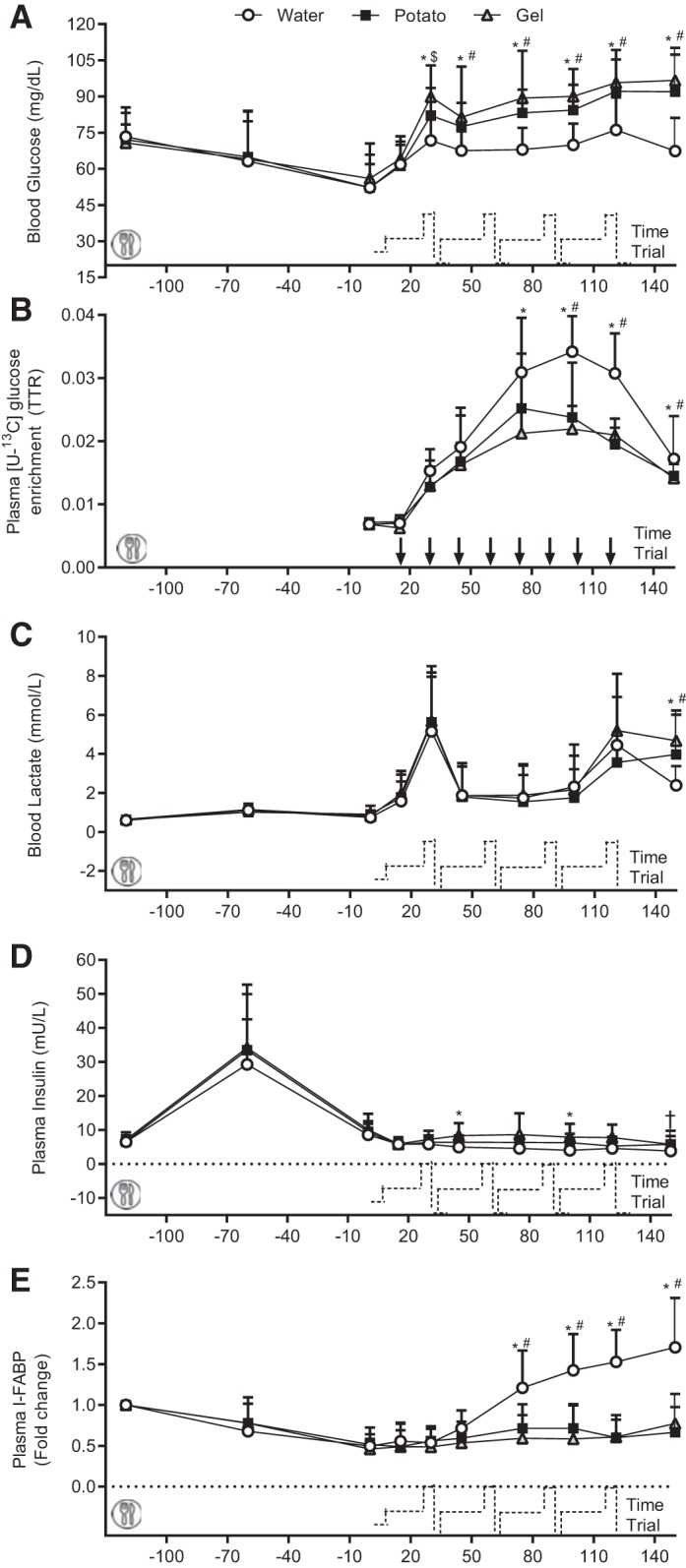
Blood glucose (*A*), blood lactate (*B*), plasma [U-^13^C]glucose enrichment (*C*), plasma insulin concentrations (*D*), and fold change from baseline of plasma intestinal fatty acid-binding protein (I-FABP) concentrations (*E*) during the experimental trial. All values are presented as means ± SD (*n* = 12). A standardized breakfast was consumed at −120 min. TTR, tracer ([U-^13^C]glucose)-to-tracee (glucose) ratio. *Significant difference between water and gel (*P* < 0.05); #significant difference between water and potato (*P* < 0.05); †tendency toward difference between water and gel (*P* < 0.10); $tendency toward difference between water and potato (*P* < 0.10).

No differences were observed in baseline measurements for blood glucose concentrations (*P* = 1.00). Blood glucose concentrations (*P* < 0.001) were elevated in both CHO conditions compared with the water condition during the cycling challenge (Fig. 3*A*). The plasma [U-^13^C]glucose enrichments were not different between CHO conditions. However, a difference (*P* < 0.001) was observed between CHO conditions and the water condition after 45 min of the cycling challenge until the end of the experimental trial (Fig. 3*B*). No differences were observed in blood lactate concentrations (Fig. 3*C*) among conditions during the cycling challenge; however, a higher lactate concentration (*P* = 0.001) was found after TT completion in both CHO conditions (potato, 4.0 ± 2.3; gel, 4.7 ± 1.3 mmol/L) compared with water condition (2.4 ± 1.0 mmol/L). Plasma insulin concentrations were higher in the gel compared with the water condition (main effect of condition, *P* = 0.003). There were no differences in plasma insulin concentrations between the potato and water conditions (*P* = 0.253). CHO ingestion reduced (*P* = 0.011) exercise-induced intestinal damage, as indicated by lower plasma I-FABP concentrations (Fig. 3*E*) in CHO conditions at 75 min of the cycling challenge, which remained lower until the end of the TT.

#### Gastrointestinal symptoms.

**Fig. 4.**
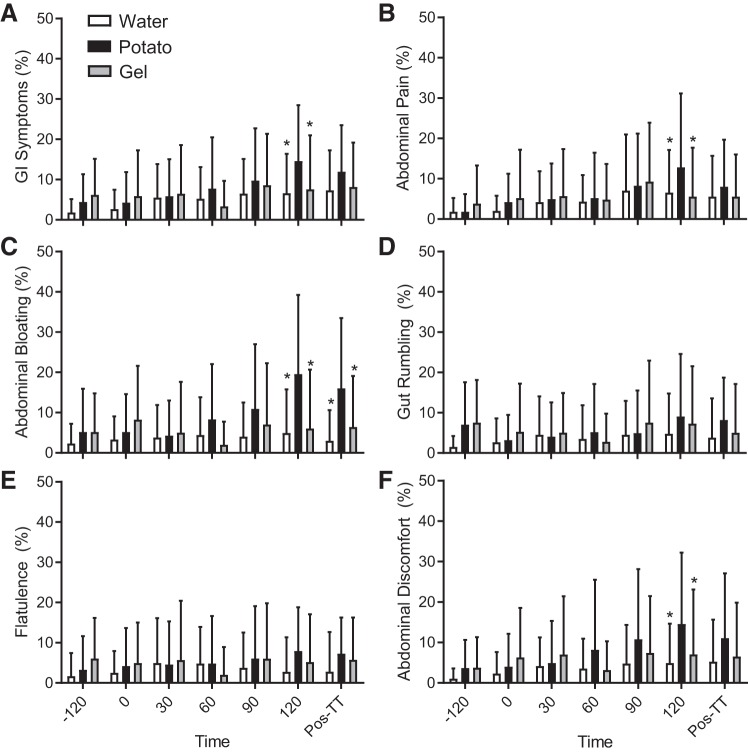
Gastrointestinal (GI) symptoms (mm) during the experimental time tria (TT). All values are presented as means ± SD (*n* = 12). *Significant difference from potatoes (*P* < 0.05).

GI symptoms are shown in Fig. 4. The overall GI symptoms were higher for potatoes than for the other conditions after the cycling challenge (120 min). Specifically, there were higher levels of abdominal pain, bloating, and discomfort during the late phases of the cycling challenge. No correlations between plasma I-FABP concentration and GI symptoms were observed.

#### Performance measurements.

**Fig. 5.**
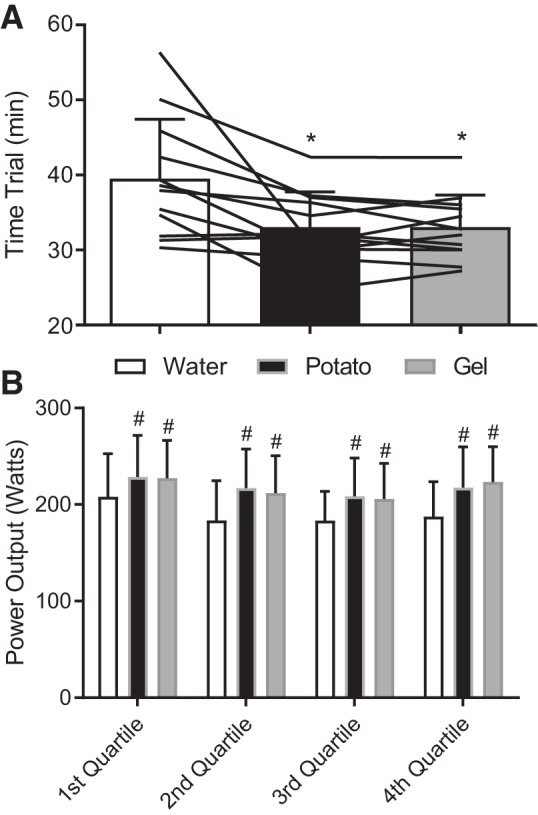
Time trial performance as total time (min) for completion (*A*) and power output during each quartile of completeness (B). Values are means ± SD (bars) and individual responses (lines) (*n* = 12). *Significantly different from water (*P* = 0.03); #significantly different from water (*P* < 0.02).

TT performance (Fig. 5*A*) was significantly faster (*P* = 0.032) in potato (33.0 ± 4.5 min) and gel (33.0 ± 4.2 min) conditions compared with the water condition (39.5 ± 7.9 min); however, no difference was observed between the potato and gel conditions (*P* = 1.00). When power output was analyzed in quartiles (Fig. 5*B*), times to completion of each quartile of the TT were statistically different (*P* = 0.02) for CHO conditions compared with water condition across all quartiles, indicating no difference in pace or race strategy selected by the athlete. In addition, TT performance was inversely correlated with blood glucose concentration [*r* = −0.72, *P* < 0.001, 95% confidence interval (CI) = −0.88 to −0.42] and positively correlated with plasma I-FABP concentration (*r* = 0.65, *P* = 0.001, 95% CI = 0.28 to 0.85) at 120 min, before the TT start.

## DISCUSSION

CHO ingestion to sustain exercise performance has been extensively studied (11, 31). However, most research has used manufactured CHO products, limiting evidence-based confirmation of whole-food sources as an effective race fuel alternative. To our knowledge, our investigation is the first to provide such a comparison of a whole-food CHO source (i.e., russet potato) to a commercially available sport food such as concentrated CHO gel in a performance-specific setting. We have demonstrated that potato ingestion during exercise exhibits similar performance improvements over water compared with the ingestion of gels during prolonged cycling in trained athletes.

The end-state vision for coaches, dietitians, and athletes is to translate research outputs into practical applications and ultimately implementation into training for more successful competition (10). The cyclists tested in this study are classified as endurance trained (13). This categorization is relevant for interpretation of results, since a significant change in exercise performance is observed only when the intervention effect is highly pronounced. In other words, the more trained the athletes, the less susceptible they are to improvement in exercise performance outcomes than a nontrained individual (22). Even with no difference in heart rate during the cycling challenge between conditions in the present study, the self-paced TT altered the heart rate response. Specifically, the potato and gel conditions resulted in an increased heart rate during the TT. This was likely due to a higher exercise intensity selection and tolerance with CHO ingestion compared with water alone. Moreover, there were no differences observed between the potato and gel conditions in heart rate, showing the ability of these treatments to reach a higher cardiovascular stimulus when 60 g of CHO/h is ingested vs. the consumption of only water.

The RPE responses were consistent with those of previous studies (33), confirming the potential of exogenous CHO in attenuating exertional perceptions during long endurance cycling (1, 33). RPE relative to watts was lower in both CHO conditions (Fig. 2), which cannot be attributed only to the effectiveness of exogenous CHO in generating more power but may also be associated with the reward value of CHO intake (43). RPE is an important marker in models of fatigue and is regularly used to dictate intensities in training sessions (16), and the similarity of the RPE/W between CHO conditions highlights the feasibility of potatoes as an alternative training or race fuel.

Proper GI function (i.e., sufficient gastric emptying rates and intestinal absorption of nutrients) is relevant to ensure the adequate delivery of fluid and carbohydrates during training and competition. Here, we showed that plasma glucose concentrations were increased to a similar extent between the potato and gel conditions vs. the water condition throughout the exercise protocol (Fig. 3*A*). Moreover, plasma [U-^13^C_6_]glucose enrichments did not differ between the potato and gel conditions, which suggests that gastric emptying rates were similar between the CHO conditions (Fig. 3*C*). Similarly, substrate utilization during the late phase of the cycling challenge demonstrated that whole body CHO oxidation rates were higher as well as fat oxidation rates being lower with the ingestion of exogenous CHO. Unfortunately, our experimental approach does not allow us to interpret the influence of food source preference on exogenous vs. endogenous CHO oxidation rates. Moreover, it has been established that the ingestion of multiple transportable amounts of CHO allows for higher amounts (90 g/h) of CHO to be consumed, thereby allowing for higher CHO oxidation rates to be achieved during prolonged exercise (23). Hence, our findings may be relevant only for ingested CHO doses of 60 g/h.

Plasma I-FABP concentrations are often used as a biomarker for gut damage in exercise studies (27, 39). I-FABP are cytosolic proteins present in enterocytes that are rapidly released into the bloodstream upon intestinal cell damage. We (29) previously demonstrated an exercise-induced increase in plasma I-FABP concentrations compared with a rested state. Importantly, previous studies have shown the potential of nutritional supplements to “protect” the gut from exercise-induced damage during prolonged exercise (26, 45), albeit inconsistently (30). Potato ingestion reduced gut damage, as indicated by similar reductions in plasma I-FABP concentrations between gel and potato vs. the water condition, throughout the exercise protocol (Fig. 3*E*). As such, more research is needed to determine optimal feeding strategies that reduce GI distress and improve gut resilience while maximizing glucose availability. Nevertheless, the present study is the first to report a correlation between exercise performance and plasma I-FABP concentrations. This highlights the importance of protecting (45) and “training your gut” (25) to reduce intestinal damage and sustain performance.

It is important to recognize that the increase in plasma I-FABP concentrations in our study was not accompanied by an increase in GI symptoms. However, the lack of correlation between GI symptoms and I-FABP is consistent with other studies (27, 39). GI symptom responses vary based on exercise mode, intensity, duration, and nutritional strategy adopted, which makes comparisons between studies challenging (34). Here, potato ingestion resulted in higher GI symptoms compared with the gel or water conditions (Fig. 4). We speculate that the higher volume of potato needed to reach the same quantity of CHO/dose of gel (i.e., 128 g/145 mL potato purée vs. 23 g/24mL gel per dose) the retrogradation (i.e., formation of resistant starch) process during cooling, which increases the indigestible proportion, could cumulatively cause higher GI symptoms in this condition. Nevertheless, average GI symptoms (Fig. 4) were lower than n previous studies (34) indicating that both CHO conditions were well tolerated by the majority of the study’s cyclists. It is worthwhile to mention that only two participants had previously chosen potatoes as their personal race fuel, but all participants regularly ingest CHO gels during races and training, and according to the gut training theory (6, 25), frequency of ingestion could also alter digestibility and perceptions of fullness. Thus, the regular use of potato purée as a race feeding strategy may reduce GI symptoms over time; however, future work would be required to confirm this assertion.

Although the higher GI distress noted in the potato condition may be explained by the higher overall volume of potatoes (~8 medium-sized potatoes) and resistant starch formation, these factors may have also influenced the significantly lower core temperature that was observed in the potato condition vs. the gel condition. The gel and potato treatments were administered at the same temperature, and there were no differences in core temperature at baseline, yet potato ingestion facilitated a 0.5°C decrease in core temperature. Indeed, our trials were conducted in ambient temperature, which differs from the majority of thermoregulation studies, which use heat and humid conditions (40); so we advise caution when interpreting core temperature observations of chilled potato purée as a cooling strategy.

Ultimately, the identification of an optimal race feeding strategy for the competition day is complex, with direct considerations like exercise mode, intensity, and duration playing a role in an athlete’s nutrition requirements (e.g., timing and dose). Furthermore, indirect considerations like taste preference, cost, and overall convenience will also influence the race fuel source. Indeed, carrying and ingesting ~1 kg of potato purée would be somewhat burdensome on an athlete; however, our approach allowed us to standardize CHO content and food consistency so that we might appropriately evaluate our study outcomes. Overall, our work simply provides a proof of principle for a whole-food source of CHO to serve as a viable sport food to be included in race feeding strategies to provide an alternative to the routine ingestion of gels during training and competition. Our outcomes can be utilized by coaches, sport dietitians, and race event organizations to incorporate potatoes as an effective performance nutrition option, with recipes being tailored to an athlete’s preference throughout training and/or a race. This will help reduce the risk of flavor fatigue (i.e., viable savory option) (28), offset financial burden, and increase diet diversity. Importantly, the nutrient matrix of a potato-sourced race fuel also contains other micronutrients that may be beneficial to improve diet quality of an athlete (5, 20).

It is worth noting that there are other investigations of periexercise food source on exercise performance. Specifically, Thomas et al. (41) observed that preexercise meals consisting of glucose, water, and lentils potentiated exercise performance in comparison with potatoes. Results comparison is limited, however, as the respective study measured performance by time to exhaustion, an impractical method with low reliability (15). Alternatively, our exercise protocol seeks to improve race day applicability, incorporating high-intensity hills during the first 2 h of exercise followed by a long cycling TT, with the total exercise duration over 150 min. Consequently, such practicality limits the comparisons between other findings. Nevertheless, the performance increase in CHO over control (i.e., water) in the present study is higher compared with use of other whole-food sources (i.e., honey) (14), CHO mouth rinse (9), and caffeine supplementation (7).

In conclusion, we have demonstrated that the ingestion of potato purée represents a viable race feeding strategy by maintaining blood glucose concentration, facilitating gastric emptying, and supporting cycling performance similarly to concentrated CHO gel products. Our results have implications for the inclusion of a whole-food-based option as a component of a race feeding strategy to support prolonged exercise performance. Future studies that investigate potato processing (e.g., baked, puréed, freeze-dried, etc.) for GI acceptance (i.e., reduced GI symptoms and intestinal permeability) would certainly optimize evidence-based performance nutrition for endurance athletes.

## GRANTS

Funding for this research was provided by Alliance for Potato Research and Education. A. F. Salvador is supported by Coordination for the Improvement of Higher Education Personnel (CAPES).

## DISCLOSURES

No conflicts of interest, financial or otherwise, to declare by the authors.

## AUTHOR CONTRIBUTIONS

J.W.B., E.M.B., and N.A.B. conceived and designed research; A.F.S., C.F.M., R.A.A., R.M.T.C., A.R.K., A.M., S.E.S., J.W.B., and N.A.B. performed experiments; A.F.S., C.F.M., R.M.T.C., A.R.K., A.M., A.V.U., R.N.D., and L.L.B. analyzed data; A.F.S., R.N.D., L.L.B., E.M.B., and N.A.B. interpreted results of experiments; A.F.S. prepared figures; A.F.S. and N.A.B. drafted manuscript; A.F.S., C.F.M., R.A.A., R.M.T.C., A.R.K., A.M., S.E.S., J.W.B., A.V.U., R.N.D., L.L.B., E.M.B., and N.A.B. edited and revised manuscript; A.F.S., C.F.M., R.A.A., R.M.T.C., A.R.K., A.M., S.E.S., J.W.B., A.V.U., R.N.D., L.L.B., E.M.B., and N.A.B. approved final version of manuscript.
